# Unbinding Global China: Fieldnotes from a West African fishing port

**DOI:** 10.1177/20438206251388520

**Published:** 2025-10-17

**Authors:** Hang Zhou

**Affiliations:** 4440 Université Laval, Canada

**Keywords:** Fisheries, Global China, maritime studies, South–South relations, West Africa

## Abstract

This commentary engages with DiCarlo and DeBoom's unveiling of ‘Global China’ as a contested concept by drawing on my field research on China's maritime engagements in West Africa. While concurring with the need to interrogate this geographical imaginary, I caution against this field's lingering tendencies of exceptionalization and territorial fixation, and probe into how these tendencies might risk confining our understanding of the differently situated actors and relations that constitute maritime ‘Global China’.

DiCarlo and DeBoom's call to ‘identify and interrogate how Global China is used, for what purpose, by whom, where, with what effects, and based on what underlying assumptions’ (2026) could not be more timely, as the concept continues to rapidly spread across both scholarly and public spheres. Scholars from a range of disciplines, including but not limited to geography, often invoke the term without sufficiently articulating the spatial, temporal, or political premises underlying its use – as if there were a tacit and shared understanding of China, the global, and their intersection among researchers and readers alike.

Whilst the analytical meaning of the metaphor ‘path’ employed in their research remains unclear to me, the authors’ analysis painstakingly unpacks the complexities belying the apparently self-evident definition or use of Global China, revealing the multiple ‘world-making and meaning-making projects’ (DiCarlo and DeBoom, 2026) that this term both draw from and further authorize. Akin to the emergence of many other keywords, sometimes it is precisely the fuzziness of a concept that enables its popular (over-)use yet risks not warranting a fundamentally transformative research agenda. The concept of Global China might also run such a risk should we not engage in a serious reflection exercise, as the authors did, on its contemporary uses and their variant underlying assumptions.

Different from the previous generation of study on Chinese transnationalism that focused on the hybridity and heterogeneity of Chinese diasporic cultures and thereby often placed the People's Republic of China (PRC) on the periphery, the new wave of research on Global China reshifts its focus on the PRC, albeit with a central concern with the *outward* projection of its state and people onto the global stage (Matin, 2024). This burgeoning body of research did not emerge in a vacuum but took shape precisely within a specific geo-economic and -political context, that is, the accelerated globalization of the PRC, symbolized and materialized by flagship programs like the Belt and Road Initiative (see also Cheng, 2023). Additionally, the rise of Global China research not only aligns with the outward expansion of the PRC but also reflects, in part, a pragmatic adaptation by some scholars of China to the increasingly constrained research environment within the PRC since the 2010s, leading many to study (Global) China from external vantage points.

While scholars on Global China such as Franceschini and Loubere (2022) explicitly call for ‘perceiving China as intimately entangled with global histories, processes, phenomena, and trends’, thereby advancing a ‘fundamentally relational perspective’ on Global China, much of the existing research under this label remains bound by the tendency to territorialize *China*. This renewed fixation on the PRC tends to narrow and homogenize the conceptual space for understanding *China*, especially when contrasted with earlier scholarship on Chinese transnationalism that foregrounded translocal flows and diasporic networks. In much of the current Global China research, the category of *China* is increasingly equated with the contemporary territorial state, thereby reinforcing a unitary and bounded conception of the term while reducing its historical complexity. This move becomes further solidified by the escalating United States–PRC geopolitical rivalries and deeply resonates with the long lineages of exceptionalizing China. As a result, many studies on Global China are primarily preoccupied with asking whether China's overseas practices are inherently or distinctively ‘Chinese’, a line of inquiry that risks reproducing culturalist, essentialist and state-centric assumptions. This orientation hence conduces to producing a skewed research geography on Global China, one that privileges certain vantage points and scales over others: the public over the private, the central state over provincial, local or borderland actors, overseas manifestations over the domestic conditions and heterogeneities that shape them.

When DiCarlo and DeBoom identify Othering as one of the six paths of Global China, they rightly observe that uses of Global China as ‘Other’ are prevalent in the remaining five paths. This also raises a deeper question: is Othering just one of the paths on a qualitatively equal footing with the others, or is it a constitutive dimension of Global China itself – suggesting that the very emergence of Global China as a concept may already be an act of exceptionalization?

Their intervention prompts us to reflect not only on the analytical uses of Global China but also on how it takes shape in lived, situated encounters. To probe these questions further, I turn to, in the following paragraphs, a vignette from my field research at the port of Alto Bandim in Guinea-Bissau. Here, the everyday practices of distant-water fishing, trading, and infrastructural use reveal how different actors, scales, and histories become bundled under the label of ‘Chinese’ – and how such an act of collapsing might risk obscuring the varied and layered presences of China's distant-water fishing as, to borrow Sheridan's words (2025: 477), ‘a nonisomorphic situation’.

Early in the morning, at high tide, a purse seiner edged slowly toward the concrete jetty of Alto Bandim port before finally securing its berth. The vessel flew Guinea-Bissau's national flag, yet a metal plaque bolted to its hull bore inscriptions in both Chinese and *pinyin*, marking the vessel's registration number and hinting at transnational ownership. Its arrival had not been immediate: the port's limited capacity meant it had to wait offshore until a Turkish vessel – also sailing under the Guinea-Bissau flag – completed its offloading before space was freed for it to dock.

The day's offloading was about to begin. A few dockworkers jumped aboard, preparing themselves with both Chinese and local crew members for hours of strenuous manual labor ahead. Commands and instructions rang out in both Chinese and Crioulo, delivered in sharp, hurried tones that conveyed a shared urgency: every minute counted. As the tide receded, the height difference between the vessel and the jetty would increase, making the task of offloading the catch more difficult.

A human chain soon formed between the vessel and the jetty. Those aboard heaved bins of fish up to the deck, from where others swiftly dragged them toward the two electronic scales. Each bin was weighed on the spot and quickly hoisted into one of two trucks idling nearby. In front of one truck stood a private Chinese entrepreneur – owner of an ice factory near the port – quickly jotting down the weight of each bin destined for his truck. At the second truck, two West African buyers – one Gambian, the other Senegalese – quietly observed. One took notes of the weight while the other observed closely the quality and species of fish being loaded.

Once the trucks were full, the remaining fish were wheeled over to a nearby processing facility within the port compound. At the entrance of the facility hung a slightly weathered plaque adorned with the flags of both China and Guinea-Bissau. Inscribed in the plaque was the facility's name and its financier: a Chinese state-owned enterprise that had pioneered China's distant water fishing activities since 1985. Despite shifting political ties and fluctuating diplomatic winds (i.e. Bissau's recognition of Taiwan between 1990 and 1998), it had maintained a continued presence in Bissau and was also the owner of the purse seiner moored at the port.

From the doorway of the processing plant, one could look out across the Geba River estuary toward the sea. At the far end of the opposite jetty stood the port's lighthouse, a navigational guide painted in wide green and white stripes. Midway up its structure, another sign captured the eye: the red, looping motif of a *Zhongguojie* (Chinese knotting), underscored by the bold letters *China Aid*. The emblem appeared almost grafted onto the structure, an unmistakable reminder of the port's transnational entanglements woven into both infrastructure and quotidian maritime life. During the inauguration two years earlier, President Umaro Sissoco Embaló had lauded this port as a testament to enduring ties, hailing them as a ‘revelation of the historical relations of friendship and heart between Guinea-Bissau and brotherly China. (Radio TV Bantaba, 2023)’. Yet, the port was not built from scratch: its first phase was financed by the African Development Bank, though no plaque or inscription now recalled this earlier history. What remained visible, etched into the built environment, were only the most recent layers of aid, defining whose ties counted in the present.

In this vignette of maritime encounter between China and Guinea-Bissau, many objects, actors, and relations could be considered as subjects of Global China. It might be tempted to add the qualifier ‘Chinese’ to the purse seiner, the fisheries catch, the trade flows, the crew members, the entrepreneurs and enterprises clustered at the dock, the processing plant, and even the fishing port itself. Yet such labeling often risks obscuring more than it illuminates. The very attempt to pin down a singular ‘Chinese’ identity to these multiple entanglements exposes their ambiguity.

Consider the vessel itself: it flew the national flag of Guinea-Bissau, carried Chinese inscription on its hull, was crewed by both Chinese and Bissau-Guinean, and was operated by a Chinese state-owned enterprise. Which of these markers should be taken as definitive of its ‘nationality’? This hybridity defies neat categorizations. Should it be viewed as evidence of a uniquely ‘Chinese’ mode of operation, even though we observe ‘second-generation’ arrangements are in fact relatively common practices in many coastal states (Campling, 2025), visible, for example, in the activities of the Turkish vessel that docked before the purse seiner.

The same difficulty arises when considering the marine capture itself. Should the purse seiner's haul be categorized as a ‘Chinese catch’ as Guinea-Bissau's? Such a question points to the limits of framing marine resource flows through strictly national-territorial lenses (Vandergeest et al., 2025). The circulation of the catch adds further complexity: a private Chinese entrepreneur and West African buyers were all present at the dock to purchase fish, which would later be distributed across their own – most likely, intra- and trans-regional – networks of traders, processors, and consumers that are neither strictly Chinese nor purely local.

Grouped under the umbrella of ‘Chinese’ are indeed qualitatively different actors whose positions and interests are anything but uniform. For instance, Chinese crew members on the purse seiner may not be embedded in the same kinds of labor relations as their land-based colleagues in the processing facility. The operator of both the vessel and the processing plan is a state-owned enterprise, but the owner of the nearby ice factor – a fish trader himself – is a private individual. While local Guineans at port called them both *chinês*, should they be analytically subsumed under the single banner of ‘Chinese capital’? Or do the divergences, collaborations and maybe frictions between them matter for how we understand China's maritime presence in this port?

With a bold emblem of *China Aid*, the fishing port of Alto Bandim also bears visible signs of Chinese involvement, but does it automatically make it a ‘Chinese’ port or project? Can such material makers be easily taken as evidence of what the president acclaimed as ‘the historical relations of friendship and heart between Guinea-Bissau and brotherly China’? Or do they risk obscuring the longer and messier genealogies of development aid and diplomatic vicissitudes that have long shaped the port site and, more broadly, Bissau's engagements with the world including China?

Vessels, catches, and infrastructures at this West African port defy easy categorizations. As a layered and contested formation, Global China is a bundle of differently situated actors and relations. Recognizing this complexity underscores the importance of resisting reductive framings that treat Global China as unitary, self-contained, or self-evident. Instead, scholars must foreground hybridity, ambiguity, and situatedness, acknowledging that the meanings and effects of ‘China’ in the world emerge through ongoing, heterogeneous, and multiscalar encounters.
